# Questions and Answers on the Essentials of Physiology

**Published:** 1888-05

**Authors:** 


**Questions and Answers on the Essentials of Physiology,** prepared especially for students of medicine. By H. A. HARE, M. D., Demonstrator and Instructor University of Pennsylvania, with illustrations. Philadelphia: W. B. Saunders. 1888.

